# Event‐Related Spectral Perturbation, Inter Trial Coherence, and Functional Connectivity in motor execution: A comparative EEG study of old and young subjects

**DOI:** 10.1002/brb3.3176

**Published:** 2023-07-21

**Authors:** Adam Gyulai, Janos Körmendi, Mohamed F. Issa, Zoltan Juhasz, Zoltan Nagy

**Affiliations:** ^1^ Szentagothai Doctoral School Semmelweis University Budapest Hungary; ^2^ Department of Neurology Uzsoki Hospital Budapest Hungary; ^3^ Laboratory of Bioelectric Brain Imaging National Mental, Neurological and Neurosurgical Institute Budapest Hungary; ^4^ Department of Electrical Engineering and Information Systems University of Pannonia Veszprem Hungary; ^5^ Faculty of Education and Psychology, Institute of Health Promotion and Sport Sciences Eötvös Loránd University Budapest Hungary; ^6^ Faculty of Computers and Artificial Intelligence, Department of Scientific Computing Benha University Benha Egypt; ^7^ Department of Vascular Neurology Semmelweis University Budapest Hungary

**Keywords:** aging, EEG, Event‐Related Spectral Perturbation, Functional Connectivity, Inter Trial Coherence, motor execution

## Abstract

**Introduction:**

The motor‐related bioelectric brain activity of healthy young and old subjects was studied to understand the effect of aging on motor execution. A visually cued finger tapping movement paradigm and high‐density EEG were used to examine the time and frequency characteristics.

**Methods:**

Twenty‐two young and 22 healthy elderly adults participated in the study. Repeated trials of left and right index finger movements were recorded with a 128‐channel EEG. Event‐Related Spectral Perturbation (ERSP), Inter Trial Coherence (ITC), and Functional Connectivity were computed and compared between the age groups.

**Results:**

An age‐dependent theta and alpha band ERSP decrease was observed over the frontal–midline area. Decrease of beta band ERSP was found over the ipsilateral central–parietal regions. Significant ITC differences were found in the delta and theta bands between old and young subjects over the contralateral parietal–occipital areas. The spatial extent of increased ITC values was larger in old subjects. The movement execution of older subjects showed higher global efficiency in the delta and theta bands, and higher local efficiency and node strengths in the delta, theta, alpha, and beta bands.

**Conclusion:**

As functional compensation of aging, elderly motor networks involve more nonmotor, parietal–occipital, and frontal areas, with higher global and local efficiency, node strength. ERSP and ITC changes seem to be sensitive and complementary biomarkers of age‐related motor execution.

## Graphical abstract

Event‐Related Spectral Perturbation and Inter Trial Coherence of finger tapping trials were compared to the baseline and between old and young subjects in the 300 ms time window of motor execution. As a functional compensation of aging, elderly motor networks involve more non‐motor, parietal‐occipital and frontal areas, with higher global and local efficiency, node strength.

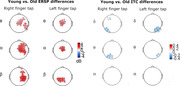

## 1 INTRODUCTION

Due to the increasing life expectancy across the world, the proportion of the adult population aged over 60 is rapidly increasing (*Ageing—Demographics*, WHO Dataset, 2022). Beside the age‐related decline in cognitive and memory functions, deteriorating motor performance piqued the interest of neuroscientists (Seidler et al., 2010). Motor dysfunction involves the central and peripheral nervous systems (Borzuola et al., 2020). Deteriorating motor performance in elderly persons manifests itself as slowing of movement (Krampe, 2002), decrease of coordination ability (Seidler et al., 2002), or as increased variability of movement executions (Cooke et al., 1989).

Aging‐related structural remodeling and selective gray matter volume reduction mostly affect changes in prefrontal, fusiform, inferior temporal and superior parietal cortices (Raz et al., 1997). Cortical volume reduction is the result of shrinkage or loss of large neurons (Haug & Eggers, 1991; Terry et al., 1987). During normal aging neuroglia cells also undergo numerous changes just as white matter integrity (Bennett & Madden, 2014; Pannese, 2021).

PET and fMRI studies of motor tasks showed overactivation of the prefrontal and sensorimotor regions for older subjects. These additionally activated brain areas can compensate for performance level despite the structural deterioration in the aging brain (Calautti et al., 2001; Heuninckx et al., 2005, 2008; Seidler et al., 2010; Ward & Frackowiak, 2003). A meta‐analysis of 40 functional brain‐imaging studies (39 fMRI/1 PET) indicates that aging‐related motor control involves not only motor cortex but also posterior brain areas such as the occipital–temporal cortex (Zapparoli et al., 2022). In addition, in a recent review of resting state PET and fMRI studies, data from eleven network measures were summarized (Deery et al., 2023) and concluded that the older brain is less modular, more integrated, and less efficient.

Oscillations in the brain regions provide a fundamental coordinating mechanism. Based on Buzsáki and Draguhn (2004), it is realistic to assume that the amplitude and/or phase synchronicity of the oscillations measured at each EEG electrode is related to the event‐related activity of the underlying cortical neurons. Connectivity information of different brain regions can provide insights into large‐scale neuronal communication and may illustrate how neural pathways are transformed and operate (Bullmore & Sporns, 2009). EEG has much higher temporal resolution than fMRI and thus has a potential to provide a detailed temporal view and show the dynamism of task‐related connectivity network changes.

We assume that EEG could confirm the previous fMRI observations further in spatial, spectral, and phase coherence domains monitored by high‐density and high‐temporal‐resolution EEG. Using the finger tap movement (button press) paradigm, as we did before in poststroke condition (Gyulai et al., 2021), could expand our understanding of electrophysiology of brain aging.

Inter Trial Coherence (ITC) is the measure of the phase synchronization of single‐trial oscillations relative to a time‐locking event, with a value between 0 and 1 (Makeig et al., 2004). Movement‐related ITC or phase‐locking index in the delta–theta frequency band is a ubiquitous movement‐related signal and independent phenomenon of movement initiation (Popovych et al., 2016). Only a few and controversial studies have addressed the phenomena of delta–theta phase‐locking value changes of movement‐related potentials in the aging brain, contradicting in whether movement‐related phase locking is an age‐dependent or ‐independent phenomenon (Liu et al., 2017; Rosjat et al., 2018, 2021).

Studying the organization of Functional Connectivity networks, which represent the synchronization of physiological signals originating from different brain regions, is a further method in the analysis of the activated motor cortex. Brain networks are organized in a highly efficient manner and can be characterized by graph theoretical metrics such as node degree, global and local efficiency (Bullmore & Sporns, 2009). While most aging studies used fMRI‐based Functional Connectivity, EEG provides unmatched capabilities for capturing the temporal dynamics of task execution.

In this study, we compared the motor execution of healthy young and elderly subjects using a visually cued finger movement experimental paradigm. For this, we focus on oscillation‐derived measures, such as the Event‐Related Spectral Perturbation (ERSP), Inter Trial Coherence (ITC), and sensor‐space Functional Connectivity. Our aims are fourfold: (1) to find differences between young and elderly motor execution‐related brain activity; (2) to characterize age‐related differences in cortical networking (3) to determine whether there are, if any, differences in bioelectric activity between young and elderly which can be used as biomarker(s) in aging; and (4) to provide data for future EEG studies, where age could be a factor in the data interpretation.

## 2 METHODS

### 2.1 Participants

Twenty‐two young (10 females, mean age: 23.6 years, SD = 3.1) and 22 elderly adults (9 females, mean age: 67.6 years, SD = 10.9) took part in this study. Clinically healthy young and elderly subjects were included in the study, who had no previous history of neurological or psychiatric problems. They were normotensive and the physical examinations were negative. They were engaged in normal daily activities.

All subjects were right‐handed according to the Edinburgh Handedness Inventory (Oldfield, 1971). The study was approved by the Ethics Committee of the National Mental, Neurological and Neurosurgical Institute. All subjects provided written informed consent before the study.

### 2.2 The experimental paradigm

Visually cued finger tapping movements were executed in the experiment. The visual cue, a black square of size 0.8 visual degree, was presented on a 22" Samsung (1680 × 1050 pixels) LCD display unit over a middle gray background. The gray level intensity of the square was adjusted linearly in a black—middle gray—black sequence in a cyclic manner with a cycle time between 7–13 s, chosen randomly for each trial (Gyulai et al., 2021). Subjects were seated in front of the display (viewing distance: 70 cm) with supported elbows, and were instructed to press a button with their right or left index finger on a custom‐made feedback panel when the contrast between the cue square and the background became zero (i.e., square indistinguishable from the background). The experiment was executed in blocks of 50 finger presses. Each block was performed using only one hand. Young subjects executed six blocks (3 blocks for each hand). Elderly subjects executed four blocks (2 blocks for each hand). Left and right hand blocks were executed alternately; the left or right hand side to start with was selected randomly by dice throwing. The possible pattern of block execution hence was either L‐R‐L‐R or R‐L‐R‐L.

### 2.3 EEG recording and data processing

**FIGURE 1:**
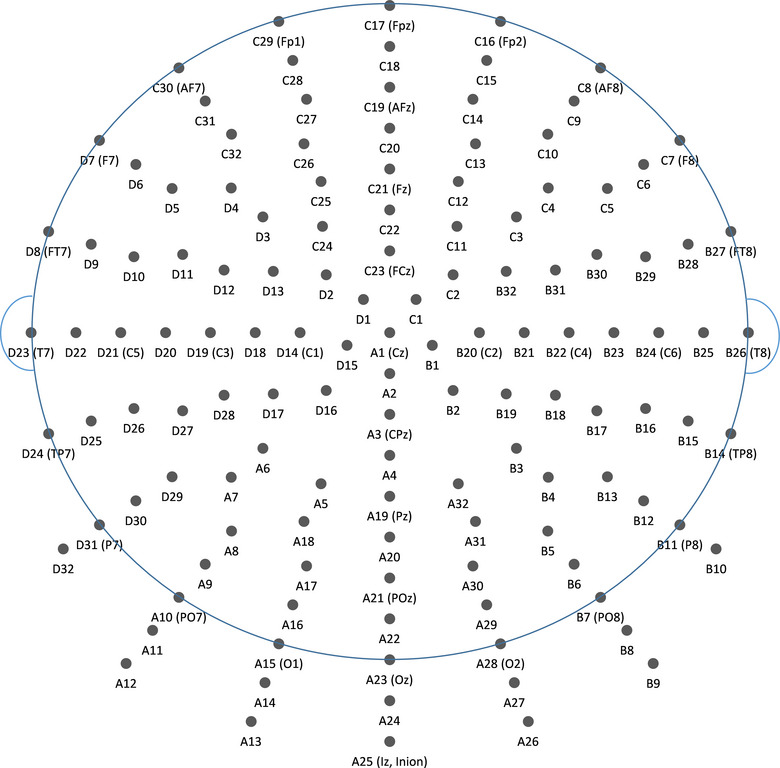
The 128‐channel Biosemi ABC electrode layout and the electrode labelling convention. In brackets, the corresponding electrode in the international 10–10 system.

EEG was recorded using a Biosemi Active Two EEG device (Biosemi B.V.) with 128 electrodes placed on the scalp according to the Biosemi ABC layout (see Figure 1 for layout and electrode labels). Data were digitized at a 2048 Hz sampling rate with 24‐bit A/D conversion. Trigger events marking the stimulus presentation and response key presses were recorded via the standard trigger port of the Biosemi EEG device.

Data processing was performed by custom MATLAB (The MathWorks Inc., Natick) scripts using EEG analysis toolboxes. Data were high‐pass and low‐pass filtered at 0.5 Hz and 70 Hz frequencies, respectively, by 4th‐order Butterworth filters, followed by a 50 Hz notch filter (*Q* = 45 quality factor) eliminating power line noise. All filtering steps were performed as zero‐phase filtering using the filtfilt() MATLAB function. EEG artifacts (blinks, eye movements, muscle noise, noisy channels) were removed using a six‐stage artifact elimination process based on Independent Component Analysis (Delorme et al., 2007; Onton et al., 2006; Weiss et al., 2016). Independent components representing identified by taking into account the results of MARA 2011(Winkler et al., 2011), FASTER (Nolan et al., 2010), and ADJUST (Mognon et al., 2011) EEGLAB plug‐ins, then removed before reconstructing the EEG signals. After artifact elimination, occasional bad channels were interpolated in the EEGLAB toolbox using spherical spline interpolation.

After the data preprocessing steps, 113 (SD = 17.1) and 88.3 (SD = 5.4) trials for young and old volunteers, respectively, were kept for each hand (left and right index finger movements) for further data processing. EEG data were then segmented into [–4500 to 2500 ms] response‐locked epochs using the button press event for trial synchronization, and downsampled to 256 Hz sampling rate. To reduce volume conduction effects, scalp current source density estimate was computed using the spherical spline Laplacian method (Perrin et al., 1989) in the Scalp Current Density Toolbox (Kayser & Tenke, 2006) with default parameters (unit sphere radius; the maximum degree of Legendre polynomials: 10; spline flexibility: *m* = 4; smoothing constant: $\lambda =10^{-5}\;$). The time‐frequency decomposition of the Scalp Current Density data was performed with complex Morlet wavelet transformation (Kronland‐Martinet et al., 2012) using the MATLAB cwt function with ‘cmor1‐1’, [0.32 73] Hz frequency range and a logarithmic step ratio (f(n)/f(n−1)=1.1) settings.

### 2.4 Event‐Related Spectral Perturbation and Inter Trial Coherence calculations

Event‐Related Spectral Perturbation (ERSP) was calculated from the Morlet wavelet‐transformed data for all channels according to the single‐trial gain model method of Grandchamp and Delorme (2011). First, the baseline‐corrected single‐trial power values, $P_{k}^{\%}$ are computed for each frequency‐time point pairs as

$$P_{k}^{\%}\;\left(f,t\right)=\frac{{\left|F_{k}\left(f,t\right)\right|}^{2}}{\mu_{B}^{\prime }\left(f,k\right)},$$

where $\mu_{B}^{\prime }\left(f,k\right)$ is the mean baseline spectral estimate for trial *k* at frequency *f* and is defined as $\mu_{B}^{\prime }\left(f,k\right)\;=\frac{1}{m}\;\sum_{t^{\prime }\in B}{|F_{k}\left(f,t^{\prime }\right)|}^{2}$, where *m* is the number of samples in the baseline interval *B*, and $F_{k}\left(f,t^{\prime }\right)$ is the Morlet‐transformed data sample at the $f,t^{\prime }$ frequency‐time value pair. The baseline interval was set to [–3500 to –3000] ms interval relative to the onset of the button press. The time interval was selected to minimize the effect of Bereitschaftspotential onset related to the button press.

*ERSP* was then computed for each frequency band—delta (1–4 Hz), theta (4–8 Hz), alpha (8–15 Hz), beta (15–30 Hz), and gamma (30–70 Hz)—using the entire trial as the log‐transformed average of the mean single‐trial power values defined as

$$ERSP_{TB-log}\;\left(f,t\right)=\;10\;\log_{10}\left(ERSP_{TB-\%}\left(f,t\right)\right)=\;10\log_{10}\left(\frac{1}{n}\sum_{k\;=\;1}^{n}P_{k}^{\%}\left(f,t\right)\right).$$

Inter Trial Coherence (ITC) is a descriptive statistical measure characterizing the circular variance of event‐related phase information or in other words, the phase consistency across trials (Makeig et al., 2004; Tallon‐Baudry et al., 1996). It is defined by the magnitude of the vector average of the oscillatory phases at every point of the time‐frequency‐channel domain across the trials. A 0 value represents random phase distribution, whereas a value 1 represents identical phase values in all trials. ITC is calculated from the complex Morlet wavelet‐transformed epoch time‐frequency information with the circ_r() function of the CircStat MATLAB Toolbox as described in (Berens, 2009).

In a previous study (Gyulai et al., 2021), we showed that baseline‐related ERSP and ITC values are at their maximum in the 300 ms interval centered around the finger movement onset, in connection with the finger tap execution. Consequently, ERSP and ITC values of this 300 ms interval were used for comparing the motor execution of old and young subjects.

### 2.5 Functional connectivity calculation

Sensor‐space Functional Connectivity was calculated based on the weighted debiased Phase Lag Index (dwPLI) (Vinck et al., 2011) using custom scripts in the FieldTrip toolbox (Oostenveld et al., 2010). dwPLI characterizes phase synchronization of oscillations across trials and is less sensitive to zero‐phase connectivity, that is, volume conduction generated spurious connections, than other phase‐based connectivity metrics, such as the phase‐locking value (PLV). Connectivity was computed for each EEG frequency band in the sensor space for a 300 ms time window centered at the time of the finger tap press. Sensor space was used to avoid potential source localization error induced inaccuracies and performing MRI scans of the subjects. The resulting weighted connectivity association matrices in which each entry represents the connection strength between two electrodes were then subsequently processed with the Brain Connectivity Toolbox (Rubinov & Sporns, 2010) to compute connectivity graph measures such as *global* and *local efficiency* and *node strength* (Bullmore & Sporns, 2009; Chennu et al., 2014; Edmunds et al., 2019).

Global efficiency represents the integration property of a network; the efficiency with which different areas can transfer information to one another. This metric is the inverse of the average shortest path length from one node to all other nodes. Local efficiency, on the other hand, represents the local information processing capability of the network, the “strength” of connectivity of a local node neighborhood or cluster. Node degree (or node strength in a weighted network) represents the number (or sum of weights) of the connections of a given node.

### 2.6 Statistical analysis

The ERSP and ITC values covering the –150 to 150 ms time and 0.5–70 Hz frequency interval were subjected to statistical analysis within subjects (baseline‐related ERSP and ITC change) and between groups (comparing old and young subjects by ERSP, ITC). To control the multiple comparison problem, the cluster‐based permutation method (Maris & Oostenveld, 2007) was used in the FieldTrip MATLAB toolbox (Oostenveld et al., 2010). Paired *t*‐tests were computed by the ft_statfun_depsamplesT() FieldTrip function. The cluster alpha level was set to a 0.001 threshold value to minimize false positives. The lower than usual threshold was selected based on suggestions for cluster‐based statistical analysis (Eklund et al., 2016). Spatial adjacency was defined by combining distance and triangulation methods from the ft_prepare_neighbours() FieldTrip function (Oostenveld et al., 2010). The threshold of distance was set to 0.4. The significance of the clusters was estimated based on 10,000 Monte Carlo randomizations. Clusters with *p*‐value > .05 were rejected. The same cluster‐based permutation statistical analysis was used to find differences between old and young subjects. However, in these test, the paired *t*‐tests were calculated by the ft_statfun_indepsamplesT() FieldTrip function. All other parameters were identical to the ones described above.

The statistical analysis of old versus young Functional Connectivity was performed in EEGLAB using the statcond() function. Since dwPLI relies on epoch‐based phase synchronization, for each subject we obtained one association matrix for each EEG frequency band, averaged from the 300 ms target interval dwPLI values, from which we computed the node strength and local efficiency values for each electrode, and one global efficiency value per subject, respectively. These values were then tested for group‐level mean differences with the nonparametric permutation test by the function statcond().

## 3 RESULTS

### 3.1 Event‐Related Spectral Perturbation

#### 3.1.1 ERSP in young adults

**FIGURE 2:**
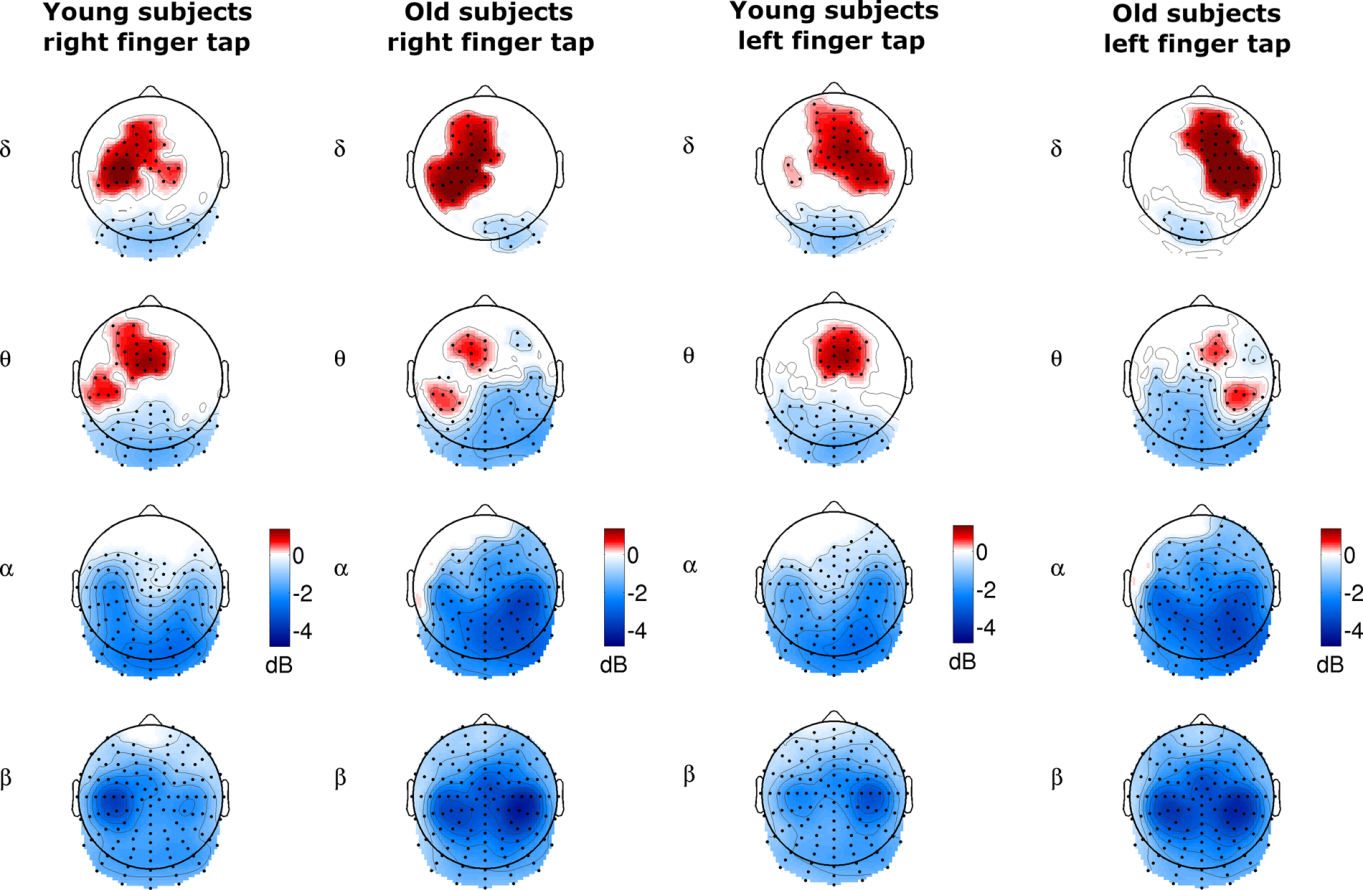
Topographic maps of averaged **ERSP** values in the 300 ms time interval centered around the onset of the button press (0 ms) compared to the baseline activity (from –3500 ms to –3000 ms). First and second columns show the right, and the 3rd and 4th columns show the left index finger movement‐related ERSP changes. Only significant areas are displayed. Red colors indicate ERSP increase while blue colors indicated ERSP decrease. Characteristic double‐peak pattern of theta activity appeared in the right and left finger taps of old subjects.

In right (dominant hand) finger movement, delta band ERSP maximum was found on the left hemisphere at electrodes D14, D17–19, D28 (see Figure 1 for electrode layout and labels). The frontal electrode D19 correlates with electrode C3 of the international 10–10 system that is considered to represent the left sensorimotor area (its cortical projection is mainly over the postcentral gyrus (69%) and—to a lesser extent—the precentral gyrus (19%)). Theta ERSP showed significant increase in the active left hemisphere with a characteristic double‐peak pattern. The anterior peak area was located midline, including electrode C23 (which is equivalent to FCz in the 10–10 system), and had larger magnitude than the second peak. Significant decrease in the alpha band ERSP was found over the entire posterior area of the brain. Beta band ERSP decreased over the entire scalp showing the largest baseline‐related change over the left central region (D17–20, D27–28) coinciding with the position of the delta band maximum peak. The cortical projection of Biosemi channels has been estimated on the basis of the formal electrode coordinates (BioSemi) and Koessler (Koessler et al., 2009) (Figure 2 column 1, positive clusters *p* = .007, SD = 0.0002; *p* = .018, SD = 0.0013, negative cluster *p* = .0001, SD = 0.0001). No significant differences were found in the gamma band.

In left finger movement, significant delta band ERSP change was found in the right hemisphere with a maximum peak area expanding over the frontal–midline area. The distribution of the alpha and beta ERSP values was similar to the right finger movement results, but the beta ERSP maximum (electrodes B17–18, B21–23) was over the right central sensorimotor region. Electrode B22 (equivalent to C4 in the 10–10 system) is over the postcentral gyrus (Figure 2, column 3, positive clusters: *p* = .0003, SD = 0.00019; *p* = .03, SD = 0.0017, negative cluster: *p* = .0001, SD = 0.0001). No significant differences were found in the gamma band.

#### 3.1.2 ERSP in elderly subjects

In right (dominant hand) finger movement, delta band ERSP maximum was found in the left hemisphere on channels above the left central–parietal and frontal–midline areas. Theta band ERSP showed significant increase in the active left hemisphere, having a double‐peak pattern similar to the one found in young subjects, but the area of the anterior maximum peak (electrode C24 in the center) is reduced compared to the young subjects. C24 is equivalent approximately to FC1 of the international 10–10 system, which is over the left superior frontal gyrus, Brodmann Area 6. The posterior theta band ERSP maximum peak was detected in electrodes D26–28, and A6–7. The most anterior electrode, D28 (approximately CP3 in the 10–10 system), is over the inferior parietal lobule (Brodmann Area 40). Alpha band ERSP decrease was detectable over the posterior part of the brain with the minimum in the right hemisphere. Beta band ERSP was observed over the entire scalp showing minimum in the ipsilateral right central–parietal region (electrodes B3, B17–19, B21–23) (Figure 2, column 2; positive cluster: *p* = .0009, SD = 0.0003, negative cluster: *p* = .0001, SD = 0.0001). No significant differences were found in the gamma band.

In left finger movement, the delta band ERSP maximum area expanded over the right hemisphere frontal–midline and prefrontal area. The double‐peak pattern of significant theta band ERSP increase was found in the active right hemisphere. The spatial distributions of the alpha and beta band ERSP values were similar to the ones obtained for the right index finger movement, but beta band ERSP minimum was also detected over the right central–parietal region (electrodes B3–4, B17, 19, B21, 23) (Figure 2, column 4; positive cluster: *p* = .001, SD = 0.0003; negative cluster: *p* = .0001, SD = 0.0001). No significant differences were found in the gamma band.

#### 3.1.3 ERSP differences of old and young subjects

**FIGURE 3:**
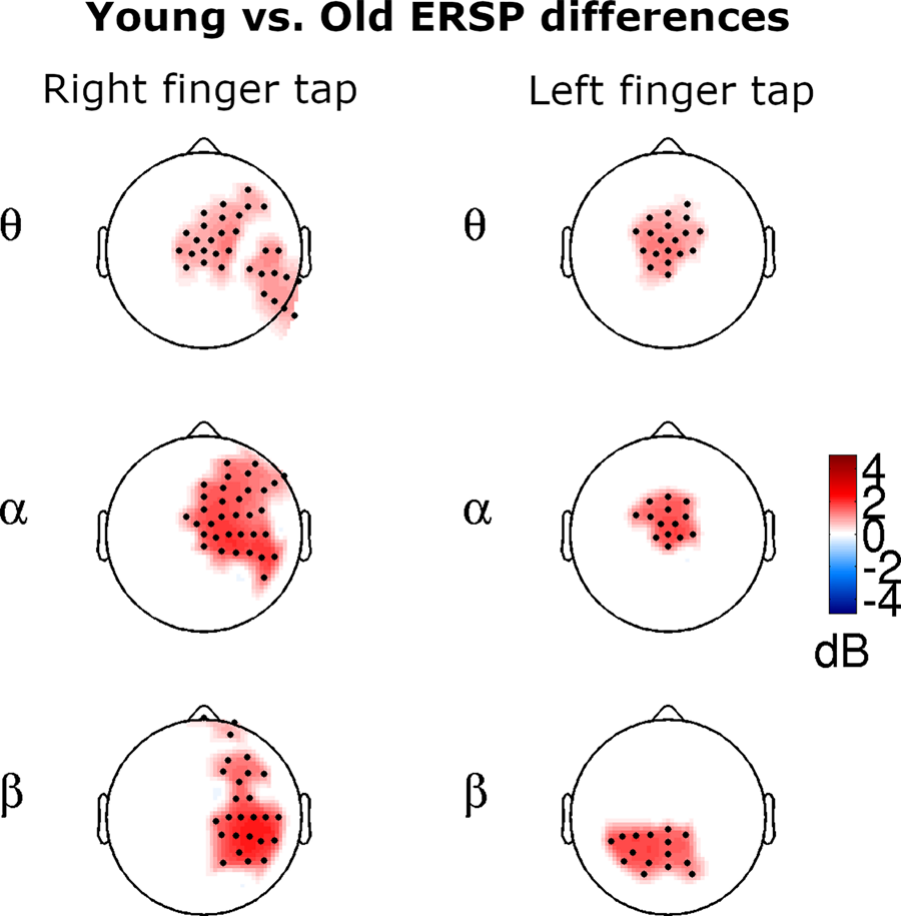
Movement‐related significant ERSP differences between young and old subjects during right and left finger movements in the 300 ms time interval centered around the onset of the button press (0 ms). Only significant clusters are displayed. Red colors indicate lower ERSP values in older subjects.

In *right index finger movements*, no significant delta band ERSP difference was found between old and young subjects. In the theta frequency band, however, elderly subjects had significantly smaller ERSP values than young ones. Theta band ERSP differences were found over the *frontal areas*, including the midline, central motor, premotor, and SMA areas. The elderly subjects showed smaller theta band ERSP values over the right prefrontal and central lateral areas of the right hemisphere. Significantly reduced ERSP values were found in the alpha band over the frontal–midline, ipsilateral right sensorimotor, premotor and prefrontal areas, and in the beta band over the right sensorimotor, parietal, and prefrontal area in the older subjects (Figure 3 column 1) (*p* = .0001, SD = 0.0001, ERSP young avg: –0.55 db, SD = 0.55, ERSP old avg: –2.389 db, SD = 1.296). No significant differences were found in the gamma band.

In *left index finger movements*, the old subjects had significantly lower theta ERSP values over the frontal–midline, central motor and premotor areas, involving the SMA in the center. In the same location but to a somewhat smaller extent, old subjects had significantly decreased alpha ERPS results (*p* = .0007, SD = 0.00026, ERSP young avg: 0.372 db, SD = 0.94, ERSP old avg: –1.390 db, SD = 1.171). In elderly subjects, significantly reduced ERSP values were found in the beta band over the ipsilateral left parietal and parietal–midline areas (*p* = .0021, SD = 0.00045, ERSP young avg: –1.639 db, SD = 0.90, ERSP old avg: –3.329 db, SD = 1.239 and *p* = .0415, SD = 0.002, ERSP young avg: –1.676 db, SD = 0.80, ERSP old avg: –3.156 db, SD = 1.251) (Figure 3 column 2). No significant differences were found in the gamma band.

### 3.2 Inter Trial Coherence

#### 3.2.1 ITC of young subjects

**FIGURE 4:**
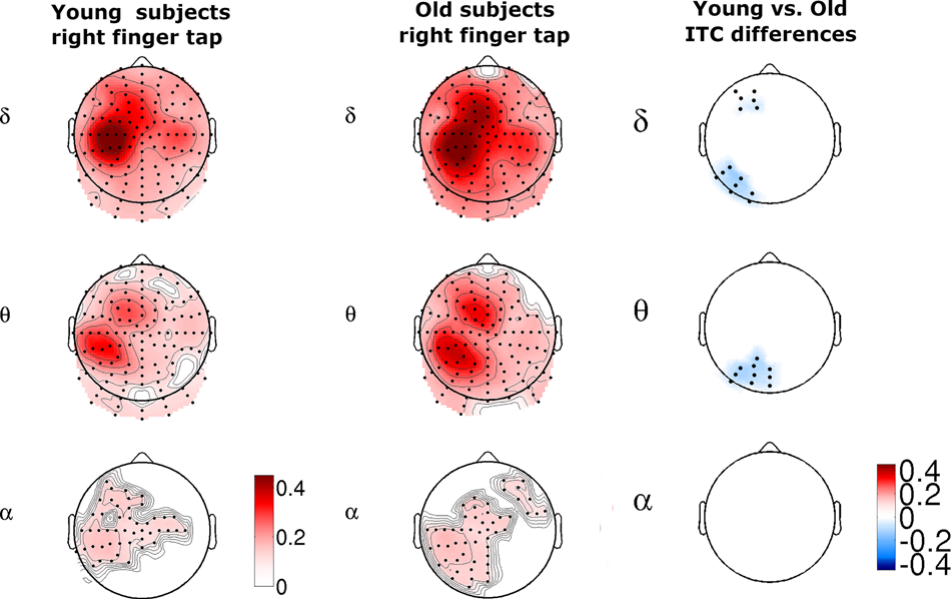
On the left: Topographic maps of averaged young and old right finger tap ITC values in the 300 ms time interval centered around the onset of the button press (0 ms) compared to the baseline activity (–3500 to –3000 ms). Only significant areas are displayed (*p* = .0001, SD = 0.0001) Last column: Right finger tap‐related ITC differences between young and old subjects. Blue colors indicate areas of significantly higher ITC values of older subjects.

In *right index finger movements*, the maximum of delta band ITC was found in the left hemisphere at the central electrodes D14, D17–20, and D27–28. Theta band ITC showed a characteristic double‐peak pattern over the active contralateral hemisphere. The contralateral theta band posterior ITC maximum was found at the electrode group of D17, D27–28, and A6. Electrode D28 (approx. CP3 in the 10–10 system) is over the inferior parietal lobule. The center of the anterior theta ITC maximum was at the electrodes C24, C23, C25, D2, and D3. Weak but extended alpha band ITC and a smaller area of beta ITC were found over the left frontal, parietal and central areas (positive cluster *p* = .0001, SD = 0.0001) (Figure 4, column 1).

**FIGURE 5:**
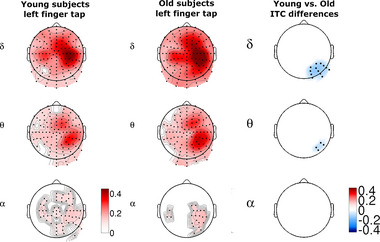
On the left: Topographic maps of averaged young and old left finger tap ITC values in the 300 ms time interval centered around the onset of the button press (0 ms) compared to the baseline activity (–3500 to –3000 ms). Only significant areas are displayed (*p* = .0001, SD = 0.0001). Last column: Left finger tap ITC differences of young and old subjects. Only the significant right parietal–occipital delta–theta clusters are displayed. Blue colors indicate areas of significantly higher ITC values of older subjects.

In *left index finger movements*, delta band ITC maximum was found over the right central electrodes B18–23. In the 10–10 system, electrode B20 corresponds to C2, whose main cortical projection is the precentral gyrus. Theta band ITC also showed a characteristic double‐peak pattern over the active right hemisphere. The smaller, anterior theta band ITC peak was observed over the medial–frontal electrodes, while the greater posterior peak was located at the parietal electrodes. The boundary zone between the two peaks (electrodes B1, B20, B21, B31) may correspond to the region of the central sulcus (*p* = .0001, SD = 0.0001) (Figure 5, column 1). No significant differences were found in the gamma band for either finger.

#### 3.2.2 ITC of elderly subjects

In right (dominant hand) finger movements, the maximum of the delta band ITC was detected at the left frontal–midline (premotor) and parietal areas. In the theta band, ITC had a characteristic double‐peak pattern over the left hemisphere, similar to the young subjects. The posterior theta band ITC peak area covered electrodes D26, 29, A6–7. The location of the significant alpha band ITC cluster corresponded to the posterior theta ITC peak (*p* = .0001, SD = 0.0001) (Figure 4 column 2).

In *left index finger movements*, the delta band ITC peak area was located over the right frontal–central–parietal electrodes B3–4, B17, B23, and B32. The anterior peak of the theta band ITC area covered more frontal electrodes. As in the right finger movement, the location of the significant alpha band ITC cluster corresponded to the posterior theta ITC peak (positive cluster *p* = .0001, SD = 0.0001) (Figure 5, column 2). No significant differences were found in the beta and gamma bands for either finger.

#### 3.2.3 Comparison of ITC between young and old

In *right index finger movements*, we found significant difference for the event‐related delta and theta band ITC between old and young subjects. We found that elderly subjects had a significantly more extended ITC area in front of and behind the left central–parietal motor region than young subjects. In the delta frequency band, significantly higher ITC was detected in elderly subjects over the left prefrontal–premotor (*p* = .014, SD = 0.0012, ITC young avg: 0.235, SD = 0.09, ITC old avg: 0.40, SD = 0.159) and parietal–temporal–occipital (*p* = .0015, SD = 0.00038, ITC young avg: 0.166, SD = 0.051, ITC old avg: 0.319, SD = 0.133) (*p* = .014, SD = 0.0012) areas. In the theta band, ITC difference (*p* = .0015, SD = 0.00038, ITC young avg: 0.166, SD = 0.051, ITC old avg: 0.319, SD = 0.133) had an occipital–parietal distribution with larger occipital extension. No significant ITC difference was found in the alpha, beta and gamma frequency bands (Figure 4).

In *left index finger movements*, elderly subjects showed significantly increased delta band ITC over the right parietal–occipital–temporal area when compared to the young subjects (*p* = .0003, SD = 0.00017, ITC young avg: 0.168, SD = 0.045, ITC old avg: 0.316, SD = 0.104). ITC difference found over the prefrontal area during the right finger movements were not found during left finger taps. The area of the posterior theta ITC difference decreased in the lateral parietal areas, spanning only electrodes B5–6 and B12–13 (B13 is equivalent to approx. P6 over Brodmann Area 39, the angular area) (Figure 5, right side).

Significant ITC differences were found in the delta and theta bands between old and young subjects. We found that the spatial extent of increased ITC values was larger in old subjects.

### 3.3 Connectivity results

**FIGURE 6:**
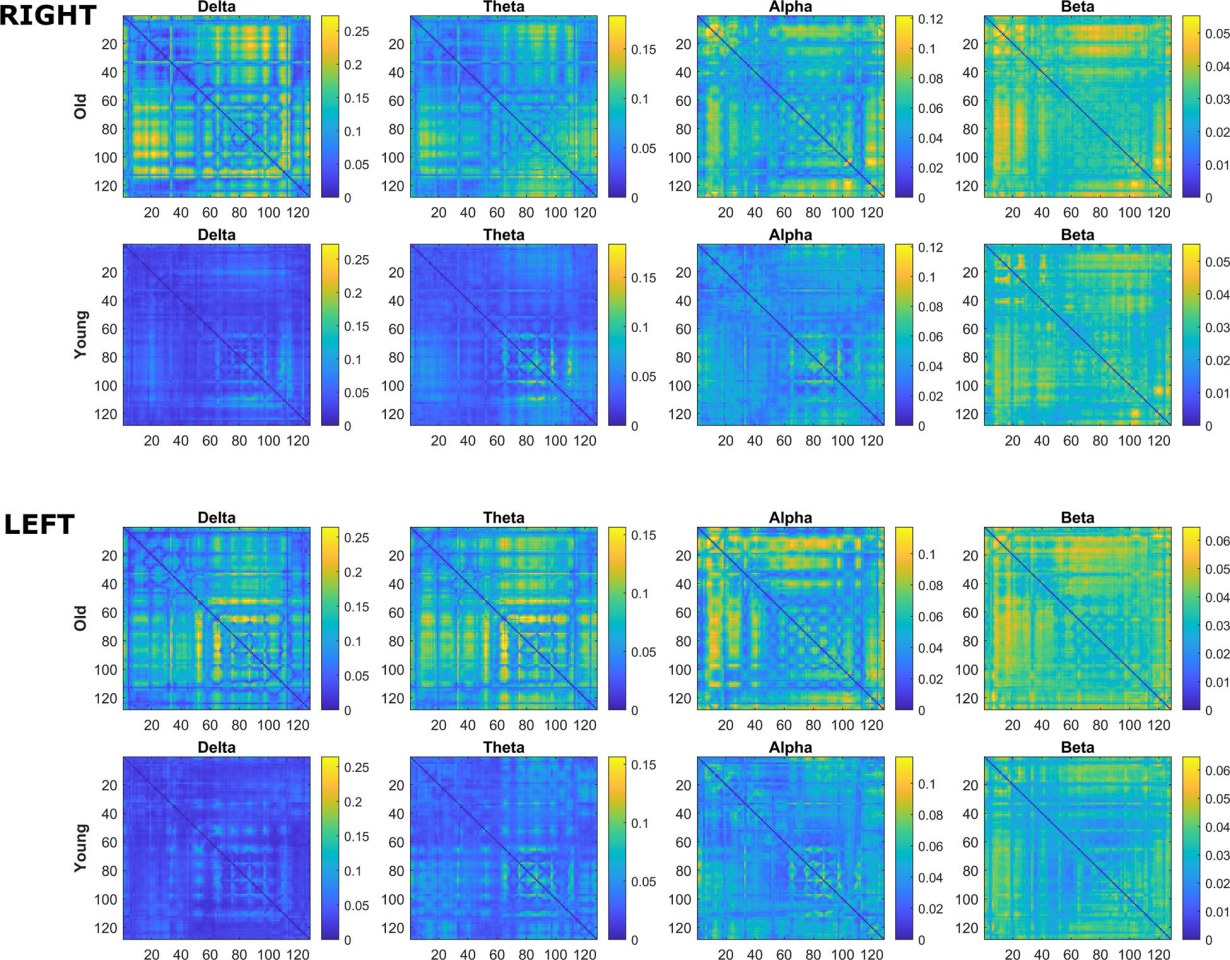
Functional Connectivity dwPLI association matrices for different frequency bands of old and young right and left finger tap executions. A time window of 300 ms was used for calculation centered around the onset of the button press. Identical scales are used in each band for the old and young group matrices to help in the visual comparison of the edge weights. The matrices show increased edge weights in the delta, theta, and alpha bands in the old group.

The sensor‐space Functional Connectivity association matrices were computed for the delta, theta, alpha, and beta bands (left and right index finger movements, respectively) based on the debiased weighted Phase Lag Index (dwPLI) connectivity method. The association matrices are shown in Figure 6. The matrices are weighted, not binary, to enable comparison of connection weight distribution differences between old and young subjects in each frequency band. For easier comparison of the old and young subject association matrices, identical weight value ranges are used for each band.

**FIGURE 7:**
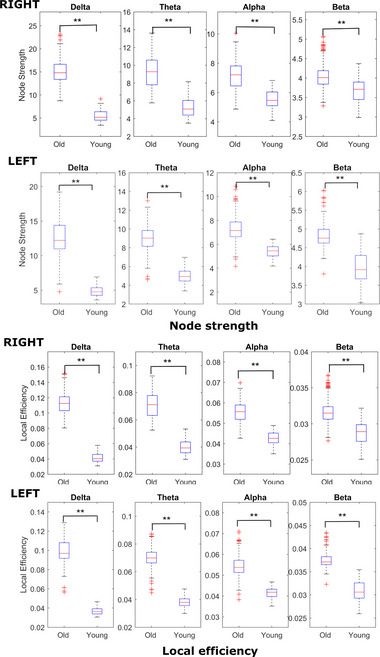
Distribution of the node strengths and local efficiency values (dwPLI) for the right and left finger tap execution. Significantly higher node strength and local efficiency values were found in all frequency bands in the old subjects.

**FIGURE 8:**
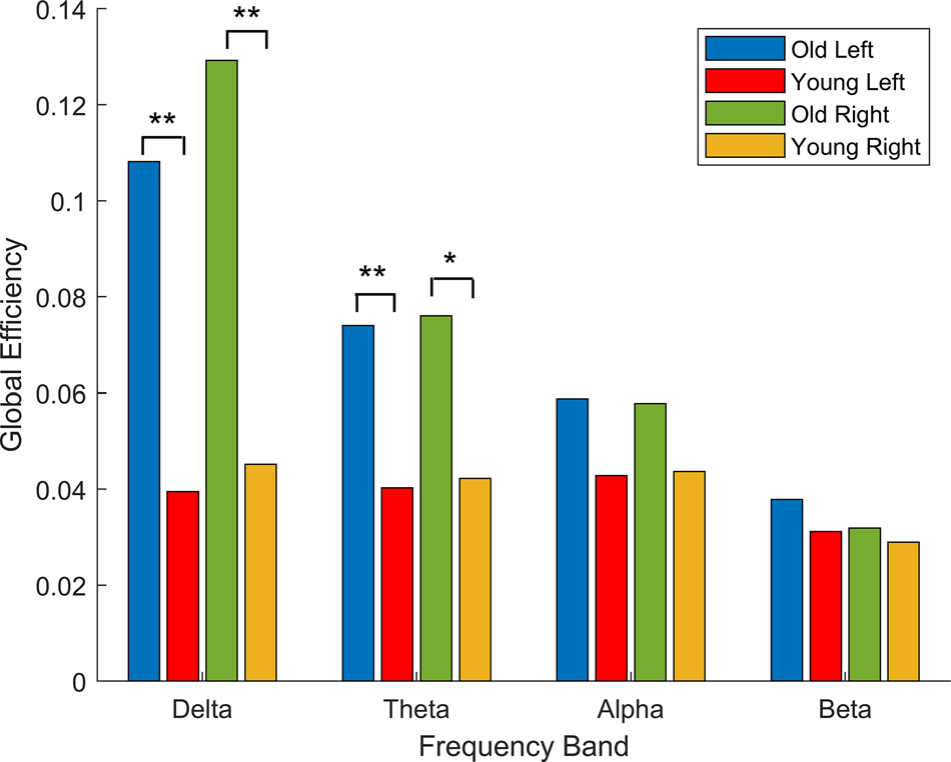
Global efficiency levels in the different frequency bands of the old and young group during left and right finger taps. Old subjects showed significantly increased global efficiency in the delta and theta bands.

We found significant differences (*p* < .01) in each frequency band between the old and young group *node strength* values, showing higher mean node strength for old subjects. Figure 7 shows the distribution of the old and young node strength values and the results of the statistical tests. The node strength metric was used instead of the node degree because we were working with weighted graphs; the node strength is the sum of all weights of the edges connected to a given node. The lower half of Figure 7 shows the distribution of the *local efficiency* values of the two groups in all four frequency bands both for left and right finger movements. Here, we also found significant differences (*p* < .005) between the two groups in the delta, theta, alpha, and beta bands, all showing higher local efficiency values for older subjects. The *global efficiency* distributions also show differences between the two age groups. Significantly higher global efficiency values were found for the older subjects in the delta (*p* < .005) and theta (left: *p* < .005, right: *p* < .03) bands (Figure 8).

**FIGURE 9:**
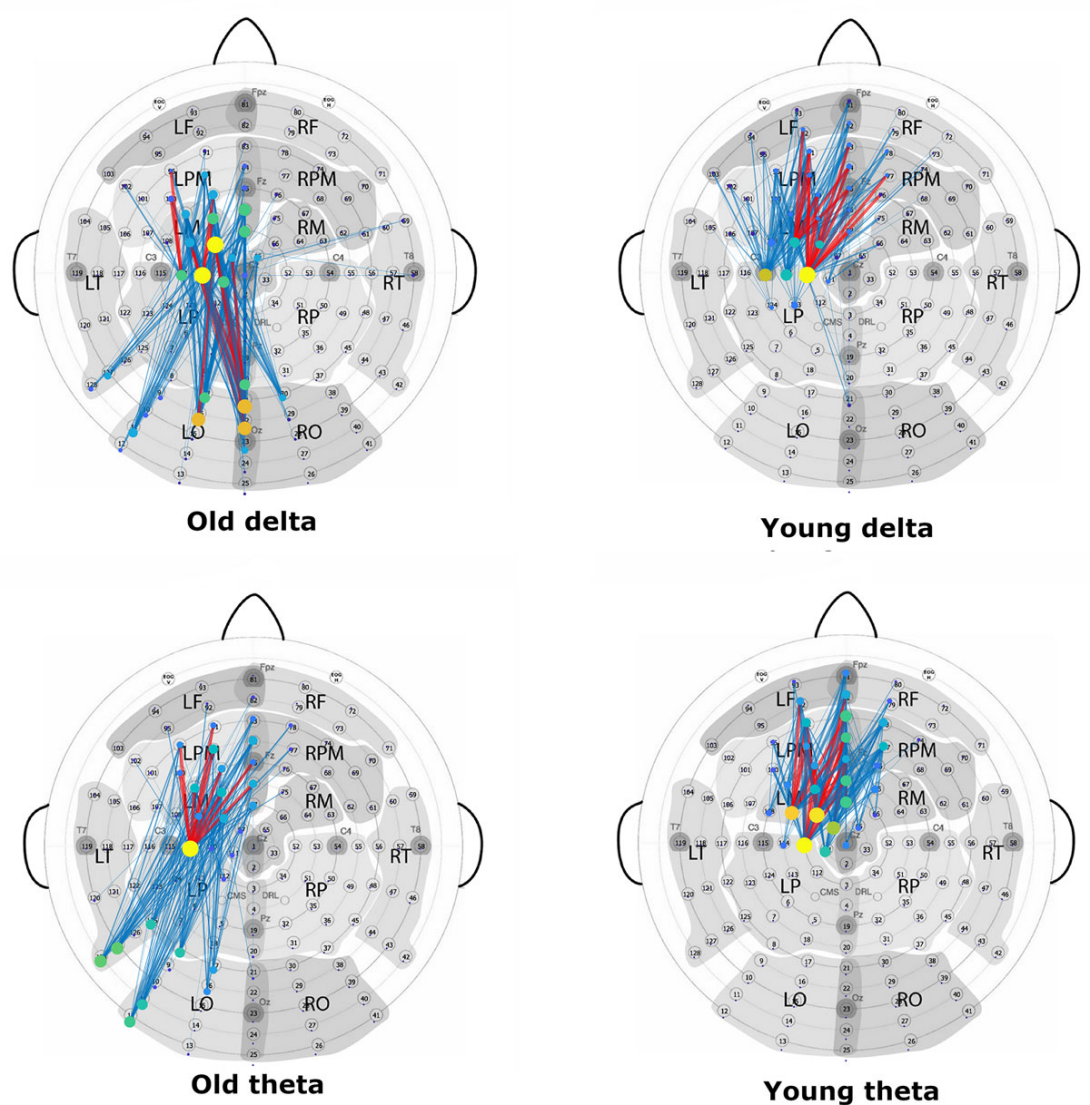
Old and young subject sensor‐space Functional Connectivity graphs of the right finger tap task in the delta (top) and theta (bottom) frequency bands. The networks of the old subjects show higher participation of the occipital–parietal areas, whereas young subjects displayed increased connections in the frontal areas.

**FIGURE 10:**
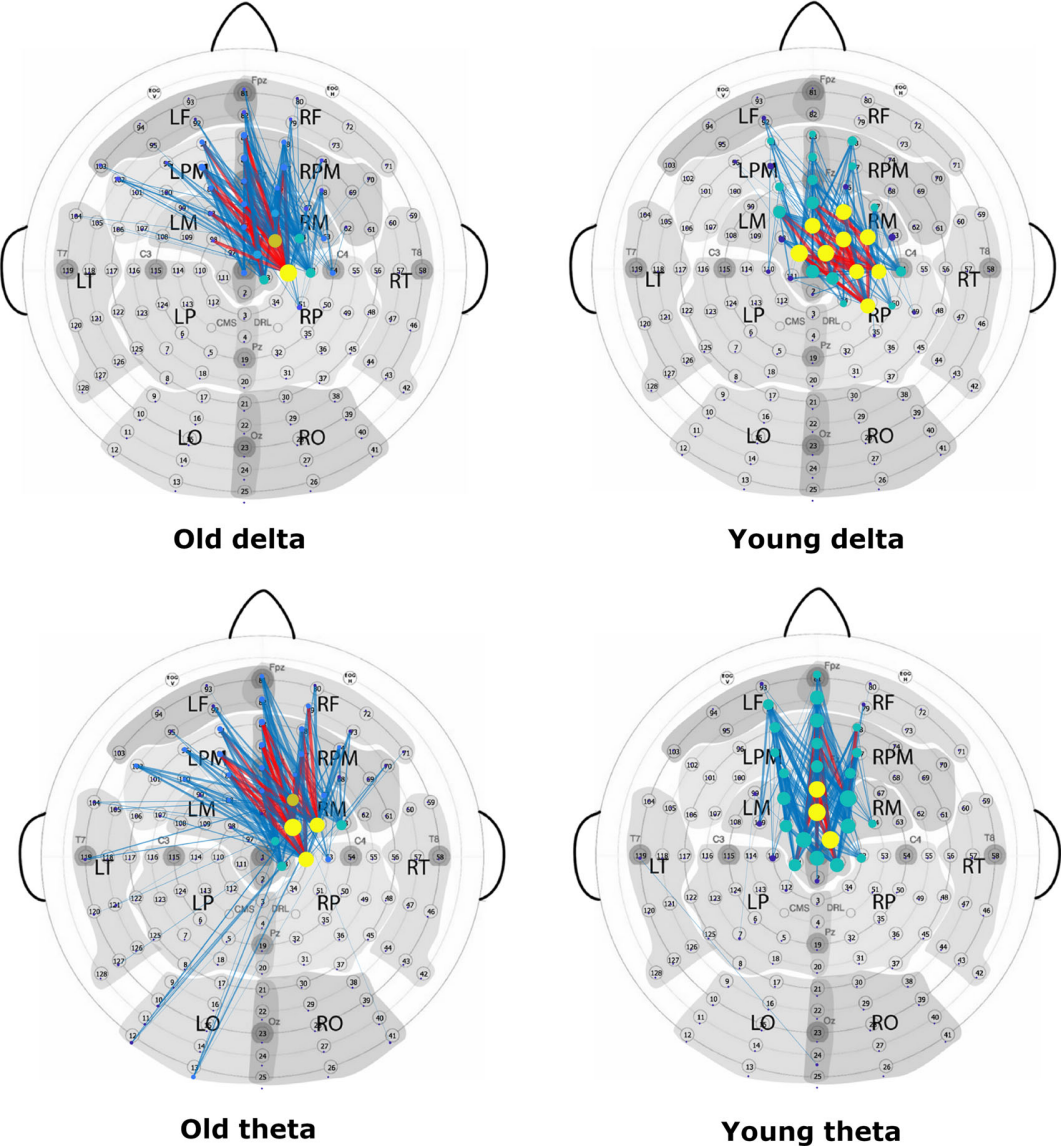
Old and young subject sensor‐space Functional Connectivity graphs of the left finger tap task in the delta (top) and theta (bottom) frequency bands. The networks of the old subjects show more extended network connections to frontal areas.

Figures 9 and 10 show the delta and theta band connectivity graphs of the old and young subjects obtained by right and left index finger movements, respectively. Proportional thresholding was used to keep only the strongest 2% of the edges in each graph. In the connectivity plots, blue lines represent the connection edges, red lines highlight the top quartile of the edges based on the edge weight. The diameter of the electrode circles represents the node strength, while the colors reflect the modularity of the graph. The connectivity analysis revealed that networks of old subjects are larger in extent both in the delta and theta bands. In the case of right motor executions, elderly subjects showed stronger connections to parietal areas, while in left motor executions frontal areas are more involved.

## 4 DISCUSSION

Normal aging brings about changes in cognitive functions and the execution of voluntary movements. Structural changes, remodeling of networks were detected and well documented by fMRI technologies. Findings on the bioelectric activity of voluntary movement in the elderly population, however, are contradictory. High‐density EEG, advanced signal detection and computation can open new avenues in understanding the field of brain oscillation and the aging process. In this study, we found that elderly subjects show significant differences in ERSP, ITC, and Functional Connectivity network measures during the execution of finger movements.

In older subjects, the execution‐related delta and theta band ITC for both hands affects the *contralateral parietal–occipital* areas. In the right finger movement execution, additionally, significant delta band ITC appears in the left prefrontal–premotor area. An age‐dependent theta and alpha ERSP decrease was observed for both hands over the *frontal–midline area*, including *SMA* in the center. In the right finger movement execution of older adults, theta, alpha, and beta ERSP decrease was found over the *right prefrontal* area. Old subjects also showed significant alpha and beta ERSP changes in right finger tap execution over the *right sensorimotor* area. Elderly subjects showed significant beta ERSP change over the *ipsilateral parietal* regions during the finger movement executions with both hands. The *global and local efficiency, node strength* were higher by both right and left button press in the delta, theta, alpha, and beta frequencies in older adults.

The finger tapping movement execution is a frequently used paradigm (Gerloff et al., 1998; Körmendi et al., 2021; Popovych et al., 2016; Urbano et al., 1996) to study the organization of the motor execution and corresponding decision‐making processes. Attention, decision‐making, motor execution, and controlling motions are associated with theta oscillations (Cavanagh et al., 2012). In the present study, we targeted exclusively the execution phase of the finger movement. It could be assumed that the synchronization and temporal organization of pyramidal, principal cells corresponding to motor execution are regulated by theta oscillations of interneurons. Cortical theta activity is suggested to be a slower variant of the thalamocortical alpha rhythms (Buzsáki et al., 2013). Hippocampal theta is linked to voluntary movement in rats (Vanderwolf, 1969). Recently, in human intracranial EEG experiments, Ramayya et al. (2021) have found that theta oscillations increased near the areas of movement‐related neural populations during movements.

In previous PET and fMRI studies, overactivation involving the prefrontal and higher‐level sensorimotor regions was found in elderly subjects (Calautti et al., 2001; Heuninckx et al., 2005, 2008; Seidler et al., 2010; Ward & Frackowiak, 2003). In the delta–theta bands, both for right and left index finger movements, elderly subjects showed a more extended significant ITC activity over the parietal–occipital regions compared to young subjects. Our finding challenges the observation of Liu's group, which concluded that no phase‐locking differences appear in the delta–theta frequency bands between old and young subjects during visually cued or self‐initiated finger tapping tests (Liu et al., 2017). They stated that phase locking in the delta–theta frequencies is a general, age‐independent phenomenon underlying the execution of simple finger movements, but the extent of synchronization between motor areas (i.e., interregional phase‐locking value) significantly differed depending on age: multiple additional intra‐ and interhemispheric connections were found in older subjects (Rosjat et al., 2018). Our study showed significant age‐related increase over the parietal–occipital and the left prefrontal areas. Our finding is in agreement with fMRI observations. In elderly subjects, increased motor‐related fMRI activation was observed in the superior temporal gyrus, supramarginal gyrus, secondary somatosensory area, ipsilateral precuneus, presupplementary motor area, predorsal premotor area, dorsolateral prefrontal cortex, as well (Heuninckx et al., 2005). A novel meta‐analysis of 40 functional brain‐imaging studies indicates that aging‐related motor control involves not only motoric brain regions but also posterior areas such as the occipital–temporal cortex (left calcarine fissure and left superior occipital gyrus, the right occipital–temporal cortex (Zapparoli et al., 2022). Calautti et al. (2001) found overactivation for older subjects in the superior frontal cortex (premotor–prefrontal junction) ipsilateral to the moving fingers. Our study found extended contralateral premotor–prefrontal delta band ITC area in right finger movements. During bimanual finger tapping employing EEG‐based dynamic causal modeling, elderly subjects showed significant couplings between the left prefrontal cortex and the left lateral premotor area (Loehrer et al., 2016). As a novelty in our analysis, we confirmed the fMRI findings with the delta–theta ITC calculation, the increase of posterior executive motor network in elderly subjects. ITC carries information on the temporal pattern of neural activity of a given neural population under the same condition (Popovych et al., 2016). Higher ITC suggests a higher involvement of cortical areas in the given task. A dynamic graph‐based approach using EEG phase locking in the low frequencies (2–7 Hz) indicated different neural information processing in older subjects; their networks displayed an overall increased connectivity, especially in motor‐related electrodes (Rosjat et al., 2021). Our results imply a growing active area working with delta–theta frequencies in older subjects. The higher global efficiency of our older subjects supports the observation of Rosjat's group.

MEG studies of visuospatial processing found that neural responses in the theta and lower alpha range (4–10 Hz) correlate with the chronological age of the prefrontal and motor cortices (Wiesman & Wilson, 2019). In a lifespan EEG study, the theta Inter Trial Coherence in the medial–frontal cortex (electrode FCz) increased from childhood to early adulthood and started to decrease from early adulthood to old age (Papenberg et al., 2013). We found that the motor execution‐related theta band ITC increased over the parietal–occipital regions in older adults, but not in the midline frontal areas (electrode FCz).

In a motor‐related theta activity EEG study, strong aging‐related theta power suppression was observed at the medial–frontal–central (FCz) region (Yordanova et al., 2020). The study suggests that this altered regulation in older subjects is due to the suppression of the medial–frontal integrating mechanism. Emerging evidence suggests that midfrontal theta oscillations are involved in cyclically orchestrating brain computations, which is more likely to be related to response execution during the tasks than to conflict processing (Duprez et al., 2020). Oscillatory midfrontal theta dynamics during reactive control mostly reflect motor‐related adjustments and the theta power is predictive of motor slowing (Kaiser & Schütz‐Bosbach, 2021). SMA‐M1 connectivity was found to be reduced in older adults with TMS (Green et al., 2018; Rurak et al., 2021) during right finger tapping.

Alpha sources in posterior brain regions were found to significantly decrease with aging (Babiloni et al., 2006). During an auditory discrimination motor task registered with 12 electrodes, widespread lower alpha amplitudes were detected in elderly subjects during cognitive and motor tasks (Dushanova & Christov, 2014).

We found significant changes in alpha ERSP in elderly subjects in the frontal–midline/SMA regions during finger tap executions by both hands. Alpha band ERSP decrease above the SMA region is a novel finding and could be interpreted as an overactivation mechanism of the aging motor network.

Sensorimotor alpha and beta rhythm changes may reflect different neural trajectories in aging (Schmiedt‐Fehr et al., 2016). Sensorimotor beta desynchronization is associated with motor preparation, execution, and imagination (Neuper et al., 2006; Pfurtscheller & Lopes da Silva, 1999). In a dominant hand grip MEG study, the increasing age was associated with increased movement‐related beta desynchronization only in the ipsilateral M1 region (Rossiter et al., 2014). In an fMRI hand grip study, reduced ipsilateral M1 deactivation was observed in older subjects at both hands (Ward et al., 2008). During right hand grips, TMS and fMRI observations suggested that the ipsilateral cortical motor areas, in particular ipsilateral M1, play a central role in maintaining performance levels with aging through increasingly facilitatory corticocortical influences (Boudrias et al., 2012). Beta band activity in sensorimotor and parietal cortex are important for accurate motor performance (Chung et al., 2017). Our beta band ERSP results confirm MEG and fMRI studies showing age‐related changes over ipsilateral regions. In addition, theta, alpha, and beta band ERSP values could be used as biomarkers of aging. Delta and theta band ITC phase synchrony could also be a further age‐related marker.

The aging brain shows that decreased efficiency of individual functional areas and that these changes are related to changes in connectivity and the small‐world architecture (modular structure) of the brain networks (Goh, 2011; Rakesh et al., 2020). Graph theory provides a suitable framework to study the integration (global efficiency), and segregation (local efficiency) properties of the connectivity network. Chong et al. (2019) performed graph theoretical connectivity analysis based on fMRI data and found decreasing local and global efficiency with aging both when compared to young participants as well as in longitudinal analyses. Interestingly, most aging network analysis is based on fMRI (Sala‐Llonch et al., 2015), very little work has been done in EEG‐based connectivity analysis of the aging brain. Wang et al. (2018) performed an EEG Functional Connectivity analysis of an audiovisual integration task and found increased global and local efficiency and degree in older subjects. They hypothesized that increase in efficiency metrics during a cognitive task indicate the presence or activation of more inter and intra module connections, representing less efficient execution and an increased cognitive demand in the aging brain. Our connectivity results reinforce these findings, as we have found significant increase in node strength as well as in global and local efficiency in the old group. This supports the idea that the aging brain requires stronger cooperation of different functional modules during the execution of a given task. Our findings suggest that both neural connectivity and the organization of these connections are important markers of processing efficiency and can be used in the future to characterize the aging process of the brain. EEG seems to be an ideal, low‐cost, and easy‐to‐use technology for future longitudinal ITC and connectivity studies to identify quantitative aging measures.

## 5 CONCLUSIONS

This study investigated aging effects in motor execution comparing old and young subjects. 128‐channel high‐density EEG measurements were performed using a visually cued finger tapping movement experimental paradigm. Event‐Related Spectral Perturbation, Inter Trial Coherence, and Functional Connectivity were computed from the measurement data and analyzed in different EEG frequency bands. We found significant changes in delta and theta ITC over extended parietal–occipital areas. Alpha and beta band ERSP decreased in elderly subjects over the frontal–midline and the ipsiparietal areas, respectively. ITC and Functional Connectivity network analysis confirmed that in old subjects a more extended network of cortical activation areas is involved in motor execution. These differences could be interpreted as bioelectric markers of aging. This approach may initiate further studies, where age could be a factor in the data interpretation in healthy and diseased conditions.

## CONFLICT OF INTEREST STATEMENT

The authors declare that they have no known competing financial interests or personal relationships that could have appeared to influence the work reported in this paper.

### PEER REVIEW

The peer review history for this article is available at https://publons.com/publon/10.1002/brb3.3176.

## Data Availability

The data that support the findings of this study are available from the corresponding author upon reasonable request.
